# Comparison of the Never Repair with Fibrin Glue and Perineural Micro-Suture in Rat Model

**DOI:** 10.29252/wjps.9.1.44

**Published:** 2020-01

**Authors:** Hossein Akbari, Behzad Farrokhi, Seyed-Abolhassan Emami, Mohammad-Reza Akhoondinasab, Peyman Akbari, Hamid Karimi

**Affiliations:** 1 Department of Plastic and Reconstructive Surgery, School of Medicine, Iran University of Medical Sciences, Tehran, Iran;; 2 School of Medicine, Tehran University of Medical Sciences, Tehran, Iran

**Keywords:** Axon regeneration, Nerve repair, Fibrin glue, Trauma

## Abstract

**BACKGROUND:**

Many different methods for nerve repair have been introduced. Nerve repair with micro-suture is the gold standard one; however, the use of fibrin glue is a promising method. This study compared the never repair with fibrin glue and perineural micro-suture in rat model.

**METHODS:**

Ten 3-4 month old male rats, weighting between 250-300 grams were divided into two groups. Left sciatic nerves of the rats were transected and repaired with fibrin glue (Tissucol^R^) in one group (A) and direct peri-neural micro-suture in another group (B). The time of nerve repair was compared between the two groups after 8 weeks. A biopsy from was taken from anastomosis site and the histopathological assessment was undertaken for axonal growth rate after anastomosis and compared between the two groups.

**RESULTS:**

The time of repair in group A was significantly lower than group B. Axonal growth rate was pretty similar between the two groups, and the difference was not significant. The mean (SD) time for repair of nerves with micro-sutures was 7.1 (1.5) minutes and the mean (SD) for repair of nerves with fibrin glue was 2.5 (0.5) minutes and the difference was significant. The number of calcification such as psammoma bodies was significantly higher in fibrin glue group.

**CONCLUSION:**

Nerve repair with fibrin glue was shown to be simpler and more time saving. The number of axons after the repair was not different in the two groups. We showed that fibrin glue may have more tissue reactions compared with micro-sutures.

## INTRODUCTION

Micro-surgical suturing is still the gold standard method for nerve repair. Suture materials can cause diverse tissue reactions such as tissue damages, inflammation and scar, while the inflammation may have a negative impact upon the end result. Many authors believe that precise co-aptation of nerve endings can be performed with suture and the risk of rupture in the nerve repair site is lower compared to other nerve repair methods. On the other hand, nerve repair with fibrin glue is more comfortable and time saving.^1^ The type of fibrin glue for nerve repair has not been addressed in other papers. The bovine type fibrin glue could have a reverse effect on the end results. In this study, we histologically compared nerve repair between micro-sutures and fibrin glue.

## MATERIALS AND METHODS

**Fig. 1:**
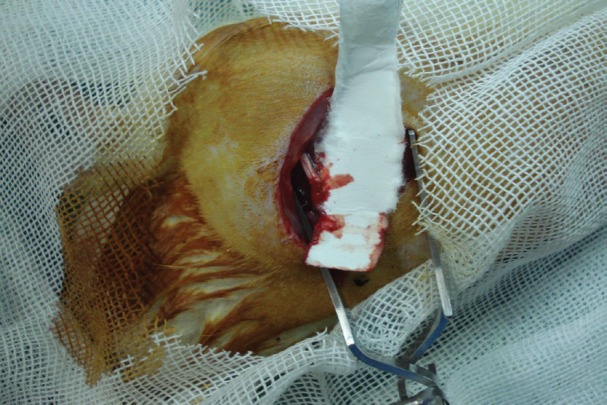
The nerve repair with fibrin glue and without suture

Ten male rats weighing approximately 250-300 g were enrolled. The experimental protocol was approved by ethical committee of our University. All surgical procedures were done under systemic anesthesia (ketamine, 5 mg/kg). During the surgery, sciatic nerve was exposed by incision on left thigh and splitting of the gluteal muscles. The nerves were sharply transected and repaired by suturing with nylon 9-0 under loupe magnification in group A and by fibrin glue in group B (bovine fibrin glue, Tissucol; Figure 1).

The time of repair in both groups was measured in minutes. The rats were kept in 12 hours cycle of day and night and enough food and water were provided for them. After 8 weeks, the rats were sacrificed with a lethal dose of potassium chloride. The repaired nerve sites were exposed and in 5 mm distal to the repair site were sharply transected. A specimen of the nerve distal to the repair sites was removed. Harvested section of sciatic nerves was immersed in a 3 percent gluteraldehyde solution and stained with Hematoxyline and Eosin (H&E).

The factors assessed include presence of inflammatory cells, number of blood vessels, fibrosis, giant cell formation and axon count 5 mm distal to anastomosis site and alignment of the fascicles.^2^ Statistical analysis of the percentage of regenerating axons was performed by student t test after testing for normal distribution of the variable.

## RESULTS

**Fig. 2:**
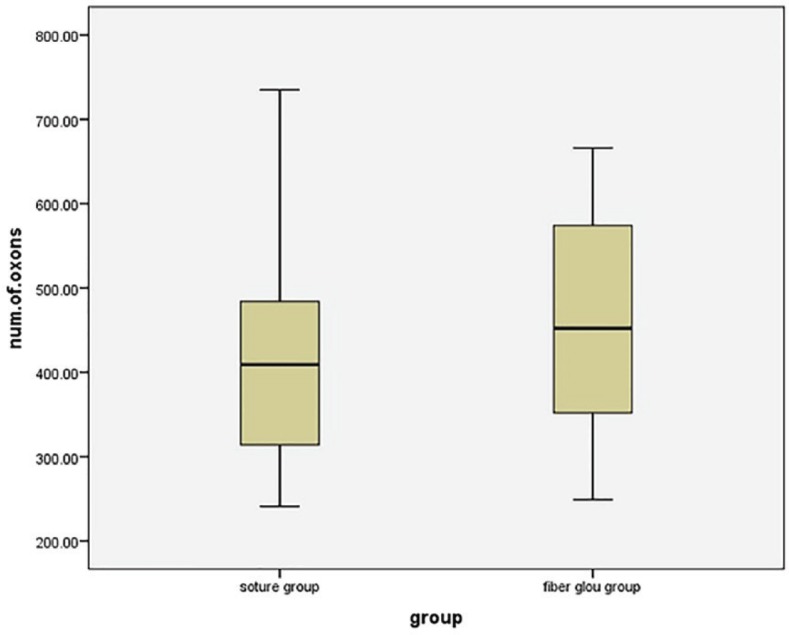
Comparing the number of axons that has been found after the repair (Those that crossed the anastomosis site).

**Fig. 3:**
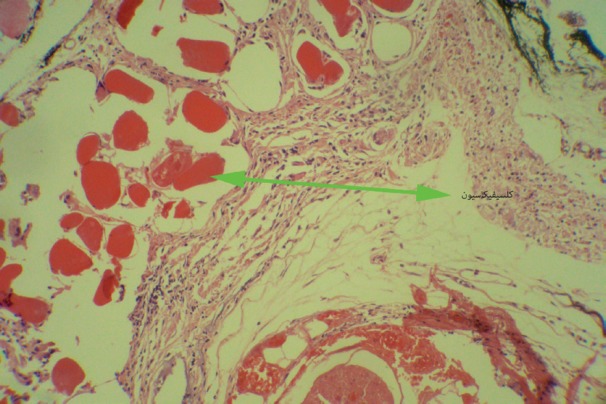
Calcifications in the repair site

In the fibrin glue group, the mean of regenerating axons was 454.1 (95% confidence interval=388.4-519.8). In the direct suture group, the mean of regenerating axons was 431.7 (95% confidence interval=355.48-507.92). The difference between the two groups was not statistically significant (*p*=0.64, Figure 2). Regarding histological assessment, the effective factors considered were, inflammatory cells, T cells, the number of blood vessels and fibrosis (Figure 2). The most important point we found was a significant greater number of foreign body giant cell reaction, peri-neural calcification and end-neural psammoma-like body calcification in fibrin glue group (Figure 3). These significant findings can be attributed to bovine fibrinogen that provoked tissue reactions that ended up with calcifications. 

## DISCUSSION

Since the time that fibrin glue was introduced in the 1970s, it has been used in several tissues.^3^^-^^5^ The use of fibrin glue in peripheral nerve co-aptation has an increasing trend. However, evidence of its benefit compared with micro-suture as the gold standard of nerve repair is scant and controversial. Crutz et.al.^6^ showed that the risk of scar formation and inflammation is more with use of fibrin glue. Among fibrin sealant groups, QUIXIL and Tissucol as common trademarks, Quixil has less inflammatory response than Tissucol in human models. This might be due to the human fibrinogen used in Quixil.

The glued anastomosis can be performed with accurate apposition and minimal trauma to the nerve. By making a fibrin semi-clot, the anastomosis can be insulated. This provides some of the benefits of sheathed anastomosis. In this way, the insertion of the scar tissue from surrounding tissue into the anastomotic site would be minimal.^7^^,^^8^ Fibrin glue has been used with conduit for nerve repairs. It could bring better results for the nerve repair with conduit.^9^^,^^10^ Also, it has been used with other repairing agents such as PRP.^11^^,^^12^

The results of nerve repair with fibrin glue with PRP were promising. In some other reports, it has been used over peri-neural suturing and it is claimed that, it will add to the strength of the anastomosis site.^13^ The fibrin glue has been used with low level laser too, and the results were promising.^14^ When it is used alone, it will provide a semi-clot around the anastomotic site. The use of fibrin glue allows proper positioning of anastomoses and repaired nerves. No torsion of the pedicle will be seen.^8^

It has been shown that the fibrin glue could stay in place in >99% of the cases. It does protect the site of anastomosis from tissue and fluid pressure.^8^ In one study, it was shown that fibrin glue repair resulted in regenerating axons travelling further into the distal nerve. It also increased the percentage of arborizing axons.^15^ The increase in arborizing axons could be another explanation for better functional and electrophysiological results after fibrin glue repair.^15^ In other studies, it was stated that fibrin glue significantly reduced surgical repair time.^16^^-^^21^ This issue has been proved in our study too. 

In 2015, Knobe *et al.* mentioned that fibrin gluing is reported to enable anatomical reconstruction with less soft tissue compromise than suture repair.^18^ In our study, it was shown that the functional evaluation and histologic assessment comparing the amount of anastomotic fibrosis, axonal regeneration, and alignment of fascicles showed that fibrin glue was as good as, if not superior to, conventional suture technique, even more calcifications have been found in the fibrin glue group. Fibrin glue was demonstrated to be faster in repairing the traumatic nerves. The rate of nerve bundle regeneration was similar to, or even better than, suture technique.

## CONFLICT OF INTEREST

The authors declare no conflict of interest.
